# Review of bone graft and bone substitutes with an emphasis on fracture surgeries

**DOI:** 10.1186/s40824-019-0157-y

**Published:** 2019-03-14

**Authors:** Hoon-Sang Sohn, Jong-Keon Oh

**Affiliations:** 1 Department of Orthopaedic Surgery, Guro Hospital, Korea University College of Medicine, 80 Guro 2-dong, Guro-gu, Seoul 152-703 South Korea; 2 Department of Orthopaedic Surgery, Wonju Severance Christian Hospital, Yonsei University Wonju College of Medicine, Wonju, South Korea

**Keywords:** Bone graft, Bone substitutes, Bone morphogenetic protein

## Abstract

**Background:**

Autogenous bone graft is the gold standard bone graft material. However, due to limitations of supply and morbidity associated with autograft harvest, various bone substitutes have been considered. This article aims to review the properties of the bone graft and various bone substitutes currently available in orthopedic surgery.

**Main body:**

Synthetic bone substitutes consist of hydroxyapatite, tricalcium phosphate, calcium sulfate, or a combination of these minerals. Synthetic porous substitutes share several advantages over allografts, including unlimited supply, easy sterilization, and storage. However, they also have some disadvantages, such as brittle properties, variable rates of resorption, and poor performance in some clinical conditions. Recently, attention has been drawn to osteoinductive materials, such as demineralized bone matrix and bone morphogenetic proteins.

**Conclusion:**

Despite tremendous efforts toward developing autograft alternatives, a single ideal bone graft substitute has not been developed. The surgeon should understand the properties of each bone graft substitute to facilitate appropriate selection in each specific clinical situation.

## Background

Bone graft procedures have been increasingly used in traumatology, tumor surgery, spine surgery, infection, and revision arthroplasty. In the US, approximately 500,000 bone graft procedures are performed annually [1]. These numbers easily double or triple on a global basis, resulting in a shortage in the availability of donor tissue conventionally used in these bone reconstruction procedures [1]. In terms of bone healing, autogenous bone graft exhibits the best osteogenic potential and is still considered to be a gold standard by many authors. Recently, another source of autogenous cancellous bone from intramedullary canal is developed. The reamer/irrigator/aspirator (RIA) technique was first developed to prepare long bones for intramedullary nail fixation [2, 3]. However, the autogenous bone graft itself is an additional operation, and complications related to bone harvesting have been reported in up to 20.6% of cases [4–7]. Disadvantages of RIA include cortical perforation, eccentric reaming, articular perforation, and intra- and peri-operative fracture [2]. Allograft has several advantages, including easy use, improved safety profiles, time advantages, availability in diverse sizes and shapes, and no donor-site morbidity. For these reasons, it is a typical alternative to autogenous bone. However, in the process of sterilization and storage, the biological and mechanical properties change, which results in loss of osteoinduction and osteogenic capability. With more demands for spinal fusion, revision surgery of arthroplasty, and joint fusion, there is a relative deficiency in allogeneic bone donors. Due to these disadvantages of autogenous bone or allograft, the necessity of bone substitutes is increasing [1].

Bone healing is a multilateral process that requires mechanical stability and revascularization along with osteogenesis, osteoinduction, and osteoconduction. Osteoinduction means that primitive, undifferentiated and pluripotent cells are stimulated to develop into the bone-forming cell lineage [8]. Osteoconduction means that bone grows on a bone surface that permits bone growth on its surface or down into pores, channels or pipes [8]. Accordingly, the most ideal bone substitute should meet such conditions, have no risk of immunological rejection (biocompatible) or disease infection, and achieve incorporation of graft in host bone by gradually being substituted by regenerated bone [8]. It should be well molded into the bony defect within a short time and should be osteoinductive, osteoconductive, and resorbable [9]. In addition, the ideal bone substitute should be thermally nonconductive, sterilizable, and readily available at a reasonable cost [9].

Bone substitutes have a diversity of composition, mechanical strength, and functional biological mechanisms. Since each bone substitute has its own unique advantages and disadvantages, the relationship between various aspects of biological properties and bone healing should be understood. In this review, we will focus on the properties of bone graft and various bone substitutes currently available in orthopedic surgery.

### Bone graft

#### Allograft

Since the autogenous bone graft might lead to complications related to harvesting and its limitation in terms of graft amount from the patient, the allograft has served as an alternative. With the development of donor screening tests, the risk of infection has been minimized [10]. The advantages of bone allograft include no morbidity of the donor-site, unlimited use of material, and availability in mechanical support with various shapes and sizes. Bone allograft is most often preserved by a freeze-drying process and vacuum-packing. However, the concern that the mechanical property of bone allograft weakens and living osteogenic cells are removed in the process of sterilization and storage are disadvantages of the allograft. These processes affect the capacity of bone healing of the allograft and result in loss of osteogenic and osteoinductive function compared with the autogenous graft. Therefore, it is mainly used in osteoconduction by providing some mechanical support.

One of the important issues of allogeneic bone is that there is potential infection risks by virus and others agents despite thorough inspection of donors and plasma examination. However, only two cases of HIV infection were reported with a risk rate of 1:1.6 million [11, 12]. Other cases of HBV, HCV and bacterial infections, including *Clostridium difficilis*, were also reported [13–16]. These types of infection cannot be removed completely even with various types of sterilization methods currently used. The risk of viral infection can be eliminated in freezing or freezing-drying process, which is the most frequently used technique in individual medical institutions [11, 17, 18]. Although a few people stressed that ethylene oxide gas can prevent viral infection, this gas is unable to penetrate cortical bone [19]. Other methods of sterilization, such as an irradiation, hydrochloric acid, and dimethyl sulfoxide, are unable to destroy a retrovirus similar to human immunodeficiency virus and feline leukemia virus [11].

The reason why the bone union rate following incorporation of allograft might be low is because the allograft has no osteogenesis and weak osteoinductivity and the process of sterilization and storage influence osteoconductivity and osteoinductivity [19–21]. Freezing or freeze-drying processes reduce the risk of an immune response of a bone graft after surgery, but sterilization itself weakens the mechanical property of a grafted bone up to approximately 50%. Furthermore, given that a large amount of gamma irradiation or ethylene oxide gas considerably decreases the osteoinductivity of bone graft, the necessity of synthetic bone substitutes has emerged to avoid adverse effects.

Allogeneic bone is available in many preparations, including morselized and cancellous, corticocancellous, cortical graft, osteochondral, whole bone segment, and demineralized bone matrix. The integration process of allogeneic bone is similar to that nonvascularized autogenous bone graft normally undergoes, but the size of allograft influences the time of incorporation. This feature is partially related to a lack of cells in the donated region for bone healing and immune reaction arising in the integration process of allogeneic bone [18, 22, 23]. In most clinical cases, allogenic cancellous bone is used to treat the partial bone defect rather than a segmental bone defect or whole-bone defect because allo-cancellous bone has no mechanical stability. Clinically, it is commonly used to reinforce spinal fusion and pack the bone defect in revision arthroplasty in particular. Two well-known types of ossification reactions, intramembranous ossification and the enchondral ossification, occur on the surface of a graft bone. An external callus is created around allogeneic bone with bridging enchondral bone formation, and resorption and creeping substitution of cortical bone occur simultaneously. Thus, the two bones are attached as if welded [24]. In addition, fusion occurs only on the junction, and dead bone trabecular mostly remains in the innermost part of a grafted bone for several years [25]. At this time, bone strength is the weakest at the 3rd to 6th month and slowly recovers during the 1st to 2nd year [24, 26].

#### Demineralized bone matrix (DBM)

DBM was first extracted from the human body in 1975. It was introduced in orthopedics in 1980 [27]. DBM is made by a standardized process originally described by Urist et al. [28, 29], in which allobone is pulverized to a minute particle size (74 to 420 μm) followed by demineralization in 0.5 N HCL mEq/g for 3 hours. The remaining acid is removed by rinsing in sterile water, ethanol, and ethyl ether. Through this process, type I collagen in cortical bone matrix and noncollagen proteins, including bone-inducing growth factors, such as BMP (bone morphogenetic proteins), transforming growth factor (TGF), insulin growth factor (IGF), and fibroblast growth factor (FGF), remain, but there is lack of mechanical support. Nevertheless, thanks to the extraction of these growth factors, DBM mainly acts as an osteoinductive material and possibly as an osteoinductive material compared with a general allograft, such as cancellous or cortical graft.

Although bone minerals are eliminated from allogenic bone, DBM is able to provide a 3-dimensional scaffold because the fibrous collagen structure of original tissues remains [30]. Since DBM is easily diluted and does not provide a mechanical packing effect for a bone defect lesion, its single use is limited, and DBM is manufactured with a variety of transmitters, including glycerol, hyaluronic acid, and calcium sulfate. Osteoinductivity of DBM is dependent on the levels of BMP-2 and BMP-7 as main growth factors. Various osteoinductivity potentials of individual DBM products are attributable to differences in DBM extraction and processing and a reduced amount of BMP in the process of sterilization and storage [31]. Although a product undergoes the same process, difference depending on the bone quality of allogeneic bone donor as a material are noted [32]. DBM has some expectations in terms of clinical use and efficacy, but research that supports its single use as a bone substitute is limited [33]. Accordingly, it is effective to add allogeneic cancellous bone or autogenous bone marrow. The most successful grafts may be composites of DBM and autogenous bone graft when used with stable fixation. DBM also has a potential risk of transmitted viral infection because it is an allogenic material. Currently, there is relevant research with a small number of randomized controlled trials. Therefore, to establish DBM as a reliable method for regular clinical use, it is necessary to produce long-term follow-up results and data.

### Bone substitutes

The most ideal bone substitute should include the ability of providing a scaffold for osteoconductivity and growth factors for osteoinductivity and should be structurally similar to real bone. The scaffold for ideal osteoconductivity should exhibit osseointegration and a 3D structure suitable for growing cells and blood vessels. In addition, it should have good biocompatibility, biodegradation, and biomechanics similar to surrounding bone tissues. Numerous bone substitutes that satisfy these conditions are commercially available in orthopedics.

#### Ceramic and ceramic composites

Ceramic bone substitutes are typical calcium-based synthetic bone substitutes that are already approved in terms of stability and effect. Given the problems with autogenous bone and allogeneic bone, osteoconductive ceramic with biodegradation draws considerable attention these days. For synthesized graft to exert its biological effects, several conditions are required: compatibility with surrounding tissues, chemical stability in body fluid, biomechanical and physical compatibility, durability in sterilization process, reasonable price, and consistency of reliable quality [34]. Today, various types of ceramic products are composed of calcium phosphate, including hydroxyapatite (HA) and tricalciumphosphate (TCP), or (calcium sulfate), or their compounds [34, 35].

Ceramic features no limitations of quantity, no risk of morbidity and infection of the donor site, and easy sterilization and storage. However, primary application of ceramics is mainly focused on bone defects, such as fracture with joint depression, because ceramics are fragile and have poor mechanical strength. Since the amount of resorption of ceramic varies depending on material, if resorption does not occur properly, it could possibly impede bone remodeling. As a result, the speed of bony union and the process of remodeling to obtain a proper strength are delayed. In addition, due to its fragility, it is difficult to mold ceramic into a desired shape during operation. The remodeling process relies mainly on ceramic biodegradability. At this time, material that is not absorbed biologically impedes the remodeling process and becomes a region of mechanical stress concentration [35]. Too slow absorption impedes bone remodeling, and too fast absorption reduces mechanical stability and causes fibrous tissue formation instead of osteogenesis [36].

#### Hydroxyapatite (HA)

HA is bioactive ceramic and a main mineral of bone. Given its density, HA with a porous structure is easily bio-absorbable and exhibits good osteoconductivity. Therefore, when it is introduced in vivo, surrounding bone tissues grows and gradually progresses through the bone substitution. Regarding the material features of HA, it can be inserted in line with a shape of a defective region. In addition, it is easily absorbed, does not generate metabolite impeding osteogenesis, and causes almost no foreign body reaction due to its excellent biocompatibility [25]. HA has very high compression and tensile strength compared with TCP. Since HA is slowly degraded and retained in vivo for a long period of time, it impedes bone remodeling extends the mechanical vulnerability of new bone, and remains as permanent stressor.

#### Tri-calcium phosphate (TCP)

Tri-calcium phosphate is osteoconductive calcium phosphate and has the most similar chemical composition to human bone. It has better absorption than hydroxyapatite (HA) [37, 38]. It is more porous than HA and features weak mechanical strength and fast absorption. More porous TCP undergoes biodegradation within 6 weeks after its introduction into the bone defect. Since its compression and tensile strength is very similar to that of cancellous bone, it is used in regions with no mechanical load [39]. Moreover, TCP has better osteoconductivity and biocompatibility than conventional bone cement with PMMA, and it is possible to inject TCP with a syringe into a bone defect or the screw insertion site in case of fracture fixation [40]. As another main component, polyphosphate is highly concentrated in osteoblasts and is involved in mineralization of bone metabolism [6, 41]. In contrast of HA, ceramic TCP is biodegraded fast in vivo. It is biodegraded within 4–8 weeks after graft, and it is difficult to obtain proper bone formation during the early period [42]. In consideration of these properties, biphasic ceramic with a mixture of HA and TCP is manufactured. Depending on the mixture ratio of these two components, it is possible to adjust the speed and degree of absorption and mechanical strength [43–46].

#### Calcium phosphate cement (CPC)

The discovery of the first CPC occurred coincidentally via the observation of calcium phosphate solubility in 1986 [47]. CPC consists of calcium phosphate. Calcium phosphate cements (CPCs) are frequently used to repair bone defects. Currently, CPC are defined as a combination of one or more calcium phosphate powders which, upon mixing with a liquid phase, form a paste able to self-set and harden in situ in the bone defect site to form a scaffold [48]. A body-temperature dissolution-precipitation reaction is one of the most important characteristics of CPC, which facilitates is ability to mold and fill the bone defect [48]. Injectability, one of the advantages of CPC, allows application of CPC in minimally invasive surgery [49]. Therefore, it is clinically used to fill metaphyseal or subchondral cortical defects caused by articular fracture. Since CPC has the material property of ceramic, bioabsorbable-enhancing additives, such as chitosan or Vicryl meshes, can be used to improve mechanical strength [50]. CPC has osteoconductivity; it is gradually absorbed in the bone remodeling process and is replaced by a new bone [39]. Currently, the paradigm has moved toward enhancement of biological interactions of CPC, such as bone tissue engineering, in addition to improvement in the mechanical strength of CPC and the addition of cells and growth factors in cement [7, 39, 51, 52]. In addition, 3D printing for fabricating CPC scaffolds is rapidly developing with a high degree of accuracy. Here, 3D printed CPC offers specific benefits for clinical applications, including easy adaptation and fixation, reduced surgical time, and good esthetic results [53, 54]. Furthermore, with recent advances in tissue engineering, “tissue regeneration by natural tissues” instead of “tissue replacement by biomaterials” has been proposed and emphasized. This new emphasis on tissue engineering is enhanced by CPC’s excellent biological interaction such as osteoconductivity, osteoinductivity, biodegradability and bioactivity [55, 56].

#### Calcium sulfate

Calcium sulfate is clinically used to fill defects, such as bone cavities, and segmental bone defect, and moreover expansion use for spinal fusion and even for filling of harvest site of autogenous bone. Through recrystallization, it becomes a solid material and gives mechanical stability to its inserted region. Calcium sulfate normally undergoes biodegradation within 6–8 weeks after its insertion into the bone defect. Given its lack of porosity, calcium sulfate has limited osteoconductivity. Given its mechanical disadvantage and rapid resorption. Compared with calcium phosphate, calcium sulfate is not often used [39].

### Bone morphogenetic protein (BMP)

Urist et al. reported that the growth factors extracted from bone organic component were able to induce osteogenesis and named them bone morphogenetic proteins (BMPs) [57, 58]. Many types of local growth factors are related to bone healing. Depending on similarities in composition, these growth factors are classified into approximately 20 multiprotein growth factor families or superfamilies. These growth factors include epidermal growth factor (EGF), insulin-like growth factor (IGF), fibroblast growth factor (FGF), platelet-derived growth factor (FDGF), and transforming growth factor (FGF). Among them, only BMPs that belong to the transforming growth factor superfamily are known to run all processes of new osteogenesis [59, 60]. After penetration of mesenchymal cells, BMPs are involved in a series of processes, including differentiation to chondrocytes, removal of cartilage, and osteogenesis. Depending on their levels, BMPs have a steep dose-response curve. If a large amount of BMPs are injected, osteoinduction occurs early, and a considerable amount of bone is generated [61]. As the injection amount increases, direct osteogenesis is increased by intramembranous ossification rather than endochondral ossification. BMP-2, BMP-7 (OP-1), and BMP-6 have similar roles and activities in the process of osteogenesis [62, 63]. To obtain the same degree of osteoinduction, a little more BMP-5 is required. BMP-3 (Ostenogenin), which is the most distributed in bone, functions as negative modulator in osteogenesis [64].

There are a few studies with a small number of randomized controlled trials only. The US Food and Drug Administration approved the use of BMP-2 for open tibial shaft fracture as a selective clinical indication [65] and the use of BMP-7 for iliac nonunion and traumatic bone defect [66]. BMP is used as an adjuvant for the spinal lumbar. When BMP-2 was used together with an allograft, its fusion rate was similar to that of autogenous bone graft [67]. These BMPs account for only 0.1% of total bone proteins and are mainly found in cortical bone. Since BMPs exist in the extracellular matrix, it is impossible to obtain BMPs until the bone matrix is demineralized [28, 68]. Accordingly, to obtain several grams (g) of BMPs, several kilograms (kg) of bones are needed. In addition, regardless of high-quality purity, they can include impurities, potentially causing unexpected reactions and results.

With the development of molecular cloning technology, these problems were solved by the creation of a large amount of recombinant human BMPs (rhBMP), which do not trigger immune reactions [69]. In the rhBMP- or bovine BMP-based animal test, these substances exhibited considerably good results. According to the research in which partially purified bovine BMPs were used for canine thoracic vertebrae fusion, the use of BMPs and autogenous bone together had the highest success rate (71%) [70]. In the thoracic vertebrae fusion of canine posterolateral transverse processes with the use of rhBMP-2, it was possible to achieve faster fusion [68]. Using an excess amount of BMPs physiologically can trigger osteolysis [71]. Depending on patients and body regions, the requirement of BMPs varies. Regarding the side effect of BMPs in the cervical vertebrae, contraindications are reported [72]. Therefore, tissue engineering approaches for long-term control and local transmission of these growth factors are a promising research area. Tissue engineering related to bone grafts have been conducted to provide all the fundamental properties of an ideal bone graft; however, it has proven difficult to achieve vascularisation in grafts which are large enough for use in clinical applications [55, 73].

## Summary

Of the graft materials for obtaining bone union, autogenous bone is the gold standard. However, there have been many shortages of autogenous bone material, and such situations are currently on the rise. To supplement autogenous bone, allogeneic bone is often used. However, depending on use conditions, the bone union rate of allogeneic bone is not satisfactory, and there is a potential for infectious diseases. For this reason, the development of synthetic materials such as ceramic has drawn a lot of attention and has been researched. Although ceramic has advantages such as easy production and no risk of infectious diseases, it has only osteoconductivity, causes mechanical vulnerability depending on material characteristics such as bioabsorption, and has difficulty playing its role continuously in the process of bone union. Currently, there is no material to completely replace autogenous bone, and it is not easy to select the best bone substitute. Therefore, in selecting the material, tissue survival capability, the size of bone defect, the size and shape of the graft material, biomechanical properties, ease of manipulation, cost, ethical issues, biological properties, and complications must be considered; biological and mechanical characteristics must be evaluated in each clinical situation.
